# COVID-19 in German Nursing Homes: The Impact of Facilities’ Structures on the Morbidity and Mortality of Residents—An Analysis of Two Cross-Sectional Surveys

**DOI:** 10.3390/ijerph20010610

**Published:** 2022-12-29

**Authors:** Benedikt Preuß, Lasse Fischer, Annika Schmidt, Kathrin Seibert, Viktoria Hoel, Dominik Domhoff, Franziska Heinze, Werner Brannath, Karin Wolf-Ostermann, Heinz Rothgang

**Affiliations:** 1 SOCIUM Research Center on Inequality and Social Policy, University of Bremen, 28359 Bremen, Germany; 2 Competence Center for Clinical Trials Bremen (KKSB), University of Bremen, 28359 Bremen, Germany; 3 Institute for Public Health and Nursing Research, University of Bremen, 28359 Bremen, Germany; 4 Leibniz Science Campus Digital Public Health, 28359 Bremen, Germany

**Keywords:** COVID-19, long-term care, nursing homes, mortality, nursing home structures

## Abstract

The COVID-19 pandemic constitutes an exceptional risk to people living and working in nursing homes (NHs). There were numerous cases and deaths among NH residents, especially at the beginning of the pandemic when no vaccines had yet been developed. Besides regional differences, individual NHs showed vast differences in the number of cases and deaths: while in some, nobody was affected, in others, many people were infected or died. We examine the relationship between facility structures and their effect on infections and deaths of NH residents and infections of staff, while considering the influence of COVID-19 prevalence among the general population on the incidence of infection in NHs. Two nationwide German surveys were conducted during the first and second pandemic waves, comprising responses from *n* = 1067 NHs. Different hurdle models, with an assumed Bernoulli distribution for zero density and a negative binomial distribution for the count density, were fitted. It can be shown that the probability of an outbreak, and the number of cases/deaths among residents and staff, increased with an increasing number of staff and the general spread of the virus. Therefore, reverse isolation of NH residents was an inadequate form of protection, especially at the beginning of the pandemic.

## 1. Introduction

In terms of infection with SARS-CoV-2 virus, nursing home (NH) residents are a particularly vulnerable group [1]. Consequently, in almost all countries which provide respective data, the ongoing COVID-19 pandemic is resulting in a particularly high death toll amongst this group [2,3]. This is especially true for the time period before vaccinations against COVID-19 were introduced. As demonstrated in an international comparison, the spread of the virus within the general population was the salient factor influencing the COVID-19-related mortality rate among NH residents in the first wave [4]. Nevertheless, differences between countries remain and can partially be traced back to the adherence to the guidance released by the European Centre for Diseases and Control [5]. The influence of facility structures has not yet been described consistently. While some international studies show that characteristics of NHs may have an impact on outbreaks among residents [6,7,8], others report that this is influenced primarily by the NHs’ environments [9].

Since the beginning of the pandemic, it has been observed that there have been sharp increases in pandemic activity in the form of waves. In Germany, the first COVID-19 wave occurred between weeks 10 and 20, 2020, and the second COVID-19 wave between week 40, 2020, and week 8, 2021 [10]. The introduction of vaccinations in Germany in late December, 2020 [11], thus, fell within the second COVID-19 wave. From April to September, 2020, it is estimated that NH residents accounted for approximately 50% of all deaths with COVID-19 in Germany [12]. Until then, for three out of four COVID-19 fatalities the Robert Koch-Institute (RKI) was able to state whether they had previously lived in a NH. Thereafter, the quality of this data deteriorated rapidly, and when for three out of four persons it could not be determined where they had dwelt, the RKI stopped publishing the rate of unknowns. The reported figures for deaths with COVID-19 among NH residents, which stood at 11,497 on 28 February, 2021 (the last day of week 8 in 2021, which marks the end of the second COVID-19 wave in Germany [10]) [13], are, therefore, a dramatic understatement of reality; analysis of claims data shows, that by the end of February, 2021, half of the 70,045 fatalities with COVID-19 could still be allotted to NH residents [14].

A national online survey conducted during the first and second wave of the pandemic revealed huge differences among individual NHs concerning the morbidity and mortality of their residents [15]. The aim of this contribution, therefore, was to analyse the data from said national survey to identify factors leading to higher or lower mortality and infection rates, and, thus, to contribute to an explanation of distinct outcomes among NHs within the German setting. The guiding research questions were: What facility structures influence resident and staff infections with SARS-CoV-2, and mortality in NHs, and which factors, besides facility structures, need to be considered? What changes are evident when comparing the first and second waves of the COVID-19 pandemic?

We first provide some basic information about the long-term care (LTC) system in Germany. We then follow up with a brief description of the data and the methods used to explore these data. After having presented and discussed the results we finish with a conclusion which also points towards further research needed.

## 2. Long-Term Care in Germany

In Germany, all citizens are covered by a mandatory long-term care insurance (LTCI) consisting of two branches: social LTCI, covering about 90 percent of the population, and mandatory private LTCI, which covers the rest of the population [16,17]. Accordingly, all individuals with long-term care needs are entitled to benefits, including cash benefits, in-kind benefits for home care, day care, respite care, and NH care. Services are provided by non-profit public and private providers, as well as private for-profit providers. Germany is divided into 16 federal states and in 2019 it had a population of 83.2 million, of which 28.5% were 60 years or older [18]. A total of 4.1 million were people with long-term care needs as defined by the German Social Code, Book XI, which regulates the LTCI, of whom every fifth (818,000, 20%) lived in one of about 11,000 NHs [19]. About 5% of all NHs are publicly run, 54% by private non-profit providers, and 41% by private for-profit providers (ibd.).

## 3. Materials and Methods

Our analysis is based on two cross-sectional surveys, carried out by the University of Bremen, from 28 April to 12 May 2020 (first wave), and from 12 January to 7 February 2021 (second wave), throughout Germany. We sent an online questionnaire developed for this study to home care providers and NHs, with automatic filtering so that only the relevant questions were activated for the respective type of provider (cf. [15] for details). The links to the surveys were sent to an opportunity sample of 7723 NHs in the first wave and 8187 in the second wave, representing around 70% of all NHs in Germany. In addition, NH organisations wrote to their members encouraging them to take part. After excluding whole data sets if not all relevant questions for this analysis were answered, 824 responses (response rate 10.7%) from the first wave and 385 responses (response rate 4.7%) from the second wave were included in the analysis. N = 142 NHs in the second wave reported having already participated in the first wave. For more details concerning data preparation and analysis see Rothgang et al. [20]. Besides questions on structural features of the NHs, the questionnaire also included clusters of questions on the incidence of the SARS-CoV-2 virus in the NHs, effects of the pandemic, e.g., on staffing and equipment, but also on changes in work processes and communication structures. Finally, respondents were given the opportunity to articulate their demands to policymakers.

An exploratory approach was used to identify factors that influenced NHs’ degree of affectedness by COVID-19. To investigate possible determinants of the target variables (self-reported number of COVID-19 cases among residents, self-reported number of COVID-19 cases among staff, and self-reported number of deaths among residents related to COVID-19), different hurdle models, with an assumed Bernoulli distribution for zero density and a negative binomial distribution for count density, were fitted. That is, two regression models were combined. In the first, a logistic regression model assessed the probability of observing at least one case in a NH. In the second model, a zero-truncated negative binomial distribution was used to model the number of cases/deaths in a facility, given that there was at least one case [21]. These models were chosen because of the excess of zeroes. To quantify the model fit the Akaike Information Criterion (AIC) of the null model was compared with the AIC of the model. A smaller AIC indicated a better model quality. All calculations were performed in R using the hurdle function of the pscl package [22,23]. In order to meaningfully assess the influence of facility size, it had to be considered that facility size also has a purely statistical influence on outbreak probability, since each resident/staff member has statistically the same probability of becoming infected independently. Consequently, as facility size increases, the probability of an outbreak in a facility increases. To test whether there was any other measurable effect beyond this statistical effect, the statistical probability of an outbreak was plotted against the actual probability. Therefore, we plotted the logarithmic frequency of observing no outbreak against the facility size. This relation should be linear if the effect is purely statistical.

First, bivariate hurdle models with one dependent and one independent variable were fitted for each covariate and outcome variable. Thereupon, all covariates that had a significant influence (two-tailed level α = 0.05) on a dependent variable were used in a multiple regression model with the same outcome variables. For explanatory variables we used the following three types of variables: (i) the characteristics of the facilities, which was ultimately what we were interested in, (ii) controls at NH level and (iii) controls at the regional environmental level. With respect to the first bundle of factors considered in the first wave we used facility size (number of beds), ownership (non-profit vs. for-profit; non-profit = private non-profit and public, profit = private for-profit) and the quantity of staff, measured as caregiver-to-resident ratio (CRR—reflects the caregiver-resident ratio, namely, if a value of 1 corresponded to one caregiver per resident, then a value of 0.6, which was approximately the mean in both waves, corresponded to 60 caregivers per 100 residents. The total number of persons was used, and not full-time equivalents). Additionally, we used the results from the questionnaire pertaining to the following measurements: staff receiving training concerning infection, prevention and control (IPC training) (binary variable), staff absence rules (binary variable; the NHs were asked if there were rules governing staff absences in case of symptoms among staff or contact with positive cases) and clinical monitoring (binary variable; the RKI made recommendations to conduct monitoring of residents for symptoms; the variable distinguishes whether these recommendations were implemented or not). In the second wave, the covariates staff absence rules, staff IPC training and clinical monitoring were not collected. Instead, the effects of the variables of frequency of testing staff, testing visitors (binary) and lack of testing (binary) were examined.

In order to minimize biases, we controlled for the type of NHs (dementia specialization (binary) and short-term care availability (binary)), but also for the spread of the virus in the general population (binary), as factors pertaining to the regional environment of the facilities. To measure the latter, the numbers of cumulative SARS-CoV-2 cases (as of 5 May 2020, and 19 January 2021 (these dates indicate the days by which half of the facilities had responded in both waves)), were, respectively, taken in relation to the total numbers of all NH residents in the respective federal state. The mean value for Germany was used to distinguish between states with below-average and above-average spread of the virus in the general population. This dichotomization was necessary because there was high heterogeneity within the states with respect to the variable, and it was unclear whether there was a linear relationship between the spread of the virus and the dependent variables.

## 4. Results

### 4.1. Description of the Samples

In both waves the responding NHs were distributed across all federal states in Germany. In the first wave they represented 7.3% of all NHs (*n* = 824 NHs) in Germany, and in the second wave 3.4% (*n* = 385 NHs). While in the first wave there were 91 NHs with cases among residents and 132 with cases among staff, both numbers more than doubled in the second wave (206 NHs and 274 NHs, respectively). At the same time, responses roughly halved, so the proportionate increase was much larger. A significant rise could also be seen in the general spread of the virus. Moreover, Table 1 reveals considerable differences among federal states, especially in the first wave (see Table 1).

In both waves, more than every third facility is a for-profit NH. Compared to national figures, these NHs were slightly underrepresented in the survey. The average number of beds was 91 in the first wave and 87 in the second. Accordingly, the NHs in the sample were slightly larger than the national average [24]. A sample description is shown in Table 2. It became clear that the samples from the two waves hardly differed with respect to most structural characteristics. While in the first wave 58% of all NHs were located in federal states with an above-average spread of the virus in the general population, this number grew in the second wave to 67%.

### 4.2. Bivariate Hurdle Models and Correlation of Covariates

The first step of the analysis was to identify variables that showed significant impact on the dependent variables through bivariate hurdle models. This forward selection was chosen because of the small sample size, especially with regard to NHs with cases/deaths in the first wave, and the high number of covariates. The results of these models for all variables with at least one significant model are displayed in Appendix A.

According to the results of the zero models, which described the probability of an outbreak within a facility, the probability of an outbreak among residents or staff, as well as deaths among residents, increased with CRR and the general spread of the virus in both waves. In addition, the first wave showed a greater likelihood of outbreaks in NHs providing short-term care as well as in NHs performing clinical monitoring. The second wave showed that facilities with problems performing rapid antigen tests were at higher risk of outbreaks among staff and residents, as well as deaths among residents. In both waves, the probability of an outbreak was higher in non-profit NHs than in for-profit NHs. With regard to the second step of the analysis, the count model, which predicted the number of cases/deaths, given that there was at least one case/death, a significant impact of variables could only be shown in a few instances. These were facility size, CRR and general spread of the virus (see Appendix A). Consequently, we included ownership, CRR, monitoring, lack of testing, short-term care, and general spread as independent variables into our multiple regression models.

With respect to facility size, when plotting the logarithmic frequency of observing no outbreak against facility size, a linear relation that would imply a purely statistical effect could not be disproved by our data (see Appendix B). For this reason, facility size was not included in the table of Appendix A. To control for this statistical effect, however, facility size was still included in the multiple models.

### 4.3. Multiple Regression Models

#### 4.3.1. Facility Structures

Concerning the variables of interest—the facility structures—the multiple models show the following results (Table 3): Facility size was an important factor with respect to the probability of an outbreak in both waves, as well as the number of cases and deaths in the second wave. It could, however, be shown that there was no discernible effect beyond statistical influence. There was no evidence of ownership influence in the first wave, but, in the second wave, in for-profit NHs the odds of an outbreak were 60% (among residents) and 57% (among staff) lower than in non-profit NHs. However, even in the second wave, ownership had no significant effect on deaths among residents; nor did the count models show any significant effect of ownership (Table 4).

When the CRR increased by 0.1, in the first wave, the odds of an outbreak increased by 15% (e^ln(OR) × 0.1^ = e^ln(4.12) × 0.1^ = 1.15) among residents and 16% among staff, while the odds for deaths among residents increased by 32% (all effects significant). In the second wave, only the odds for an outbreak among staff increased significantly by 23%.

The count models showed that when the CRR increased, the number of cases among residents and staff and the number of deaths among residents in the first wave increased as well (all effects significant). In the second wave, the number of cases among residents and staff also increased with increasing CRR (all effects significant), whereas there was no significant effect on the number of deaths among residents.

Concerning the measurements that were integrated in the analysis, it could be seen that, in the first wave, the odds for cases/deaths among residents and staff in NHs conducting clinical monitoring (only measured in the first wave) were significant and vastly higher than in NHs without clinical monitoring. With *n* = 133 there was a high number of missing values relating to clinical monitoring. To reduce the number of excluded cases, a third category was built for this variable (monitoring missing) and also integrated into the analysis. The zero models showed that there were also significant differences between NHs without clinical monitoring and NHs with missing information concerning this variable. In an additional model, to verify that the effect of monitoring is not biased due to the high number of missing values, the categories no clinical monitoring and monitoring missing were summarized. This model showed that the odds ratio (OR) for cases/deaths among residents/staff was still significantly higher in NHs with clinical monitoring, but the odds for outbreaks were, then, much lower. The count models only revealed significant effects for cases among staff. With regard to antigen tests (only measured in the second wave) the OR for outbreaks among residents and staff in NHs that had problems carrying out tests as planned was significantly higher than for those without such problems. There were, however, no significant effects with respect to the number of deaths among residents, and the count models showed no significant effect at all (see Table 3 and Table 4).

#### 4.3.2. Control Variables

With regard to the controls on the relation between the NHs and the environment, offering short-term care had no significant effect on any dependent variable in any model. Due to a high number of missing values, we added an additional category (short-term care missing) here, too, which only confirmed the results.

The influence of the spread in the general population could be seen, especially, in the first wave. In NHs that were located in federal states with an above-average general spread, the odds for an outbreak among residents and staff were more than twice as high as in NHs in federal states with a below-average general spread. The odds for deaths among residents were much higher here, as well. In the second wave, the odds for cases among staff were still significantly higher in above-average federal states, but there was no other significant influence. The count models showed that in NHs in federal states with an above-average general spread, the number of cases among residents and staff was significantly higher in the first wave.

#### 4.3.3. Model Quality and Multicollinearity

A comparison of the AIC of the null model with the AIC of the respective model showed that all models in which the covariates were included had a higher quality and the dependent variable could be explained correspondingly better with the help of the included covariates.

The analysis of multicollinearity showed that ownership was significantly associated with CRR and general spread. In both waves, it could be seen that for-profit NHs were significantly more frequently located in federal states with below-average spread of the virus (*X*^2^ = 44.6; *p* < 0.001 in the first wave and *X*^2^ = 12.1; *p* < 0.001 in the second wave) and, at the same time, had a significantly lower CRR (mean CRR in the first wave: non-profit NHs = 0.63; for-profit NHs = 0.57; t = 4.562; *p* < 0.001; mean CRR in the second wave: non-profit NHs = 0.65; for-profit NHs = 0.54; t = 4.302; *p* < 0.001). Moreover, for-profit NHs were significantly smaller than non-profit NHs in the second wave (mean number of beds: non-profit NHs = 91.4, for-profit NHs = 80.5; t = 2.508; *p* < 0.05). These analyses showed that ownership was related to CRR, facility size (only in the second wave) and general spread. For this reason, the multiple models were additionally calculated with for-profit and non-profit NHs only. Within these two groups the effects on the target variables were similar as before. For the interpretation of staffing levels, it is common practice to denote the ratio in terms of full-time equivalents to residents; here we determined this ratio accordingly. This showed that there was a strong correlation between CRR and the ratio of full-time staff equivalents to residents.

## 5. Discussion

To conclude, the multiple models showed that in both waves the probability of cases/deaths among residents and staff respectively increased with increasing CRR values and the general spread (see Table 3 and Table 4). Higher risk was also evident in facilities that conducted monitoring (first wave) and facilities that had problems carrying out antigen testing (second wave). In the second wave, the probability of cases among residents/staff was significantly smaller in for-profit NHs.

On the basis of theoretical assumptions, it could be assumed that there would be opposing effects with regard to the CRR. On the one hand, a higher CRR implied a higher number of contacts between staff and residents, as well as with other staff, and, thus, a higher risk of infection. On the other hand, a higher CRR enabled the adequate implementation of measures, such as testing and isolation. The results showed that the negative effect concerning infections and deaths was significantly greater, especially in the first wave. Similar results concerning the influence of the number of caregivers were provided elsewhere [6,7].

With regard to the general spread of the virus a comparison of the two waves showed that the influence of general spread on morbidity and mortality among residents diminished over time. At the same time, there was still a correlation with cases among staff in the second wave. It must be considered that during the first wave there were still significantly greater differences between federal states, in terms of affectedness, than in the second wave. While the coefficient of variation of the cumulative cases per 100 NH residents was 0.60 in the first wave, it was 0.36 in the second wave. Greater heterogeneity in the independent variable could lead to an increased statistical effect here.

The difference between the first and second waves can also be explained by the learning effect of staff, residents and visitors. In the first wave, they were all faced with a completely new situation, while, in the second wave, most people were familiar with the pandemic situation and adequate protection measures. Additionally, better measures against infections, like antigen tests and the availability of clinical masks, were implemented in the second wave. These could have led to fewer cases of transmission from staff to residents. This seems plausible in light of the fact that the RKI’s first recommendations for implementing protective measures were not published until 15 April [27], in the middle of the first wave. Recommendations were then continuously adjusted as the pandemic progressed [12]. Overall, a significant influence of the general spread was also evident in other international publications [4,7,8,9].

With regard to clinical monitoring, it could be assumed that there were no grounds for increased morbidity except that NHs either only started monitoring when the first cases occurred or monitoring led to an increased detection of cases. Conversely, the fact that there were increased cases in NHs that had problems with antigen testing suggested that proper testing led to a reduction in cases. Similar results for NHs in Germany were found by Tsoungui Obama et al. [28]. On the other hand, it is possible that staff in facilities in which cases occurred had less time to perform testing as directed, due to increased workloads.

In the second wave, the risk of cases among staff and residents was lower in for-profit NHs. An analysis of the independent variables showed negative correlation between ownership, CRR, and general spread. One reason for the better results of private NHs could, thus, simply be that they were predominantly located in areas with less general spread, and that their average CRR was lower than the CRR in non-profit NHs. Whether in the second wave there was an additional influence of ownership beyond these factors requires closer examination. In Konetzka et al. [7], most of the studies examined did not show any significant influence of ownership. In a few of the studies considered, better outcomes were also seen in private facilities. However, national differences, in terms of long-term care insurance systems and the role of different providers within care, must also be considered in this context.

## 6. Limitations

With regard to the first wave a comparison between an extrapolation of the number of infected residents and the official number of resident deaths attributed to COVID-19 by Rothgang et al. [15] suggested that, in respect of these numbers, there was no selection bias in the sample. Additionally, concerning the structural parameters, the samples from both waves were comparable to the national average. There were, therefore, no indications for structural bias, though this could not be completely ruled out. Especially with regard to the count models, the results of the first wave should be interpreted with caution, since only a few cases occurred here at all. Furthermore, the analysis only took the facility level into account. There are several relevant resident-level factors [29], as well as individual factors concerning the staff, such as educational level [30] and organisation of staff [8], that could not be included in the models, due to missing data. The effectiveness of measures could only be examined to a limited extent. More detailed studies are needed, including study designs that are suitable for making cause-and-effect statements. As a strength of the study, it must be emphasized that the data allowed a comparison between the first and second waves. This allowed the analysis of the different situations during the pandemic and provided information on its development.

## 7. Conclusions

The results reveal, at least at the beginning of the pandemic situation, a large influence of the general spread to cases and deaths in NHs independent of other factors, thus establishing that reverse isolation of NH residents is an inadequate form of protection and, rather, that a reduction in prevalence in the general population is indicated. The facility structures that exerted an influence on cases and deaths among residents could not be influenced in the short term, or only to a very limited extent. With respect to the CRR, it is important to keep in mind that the results do not suggest reducing staffing levels, even if this would have been beneficial in terms of the likelihood of outbreaks during the pandemic, because a higher CRR increases possibilities for infection control and quality of care. For this reason, measures must be found that allow a higher deployment of staff without harming residents during a pandemic.

Overall, it should be reiterated that the present study dealt with the situation in German NHs at the beginning of the COVID-19 pandemic, i.e., during the first two waves. For this reason, the findings to be drawn from the results are not generalizable to every other phase of the pandemic. However, the analysis allows a retrospective assessment of the handling of the pandemic in general and, to a limited extent, of the measures implemented. Accordingly, the results can be consulted in similar situations in the future to find a better response to comparable problems.

## Figures and Tables

**Table 1:** Distribution of the sample according to federal states.

	Responding NHs (NHs with an Outbreak among Residents; Staff)		Number of NHs in December 2019 ^a^	Cumulative Cases per 100 Residents in NHs According to Official Statistics ^b^	
	1st Wave	2nd Wave		1st Wave	2nd Wave
**Missing Values**	***n* = 5**	***n* = 1**	**-**	**-**	**-**
Baden-Wurttemberg	116 (20; 33)	27 (20; 22)	1519	36	303
Bavaria	130 (21; 33)	96 (48; 80)	1522	39	336
Berlin	22 (3; 3)	8 (5; 7)	299	21	392
Brandenburg	26 (2; 1)	17 (9; 10)	339	12	242
Bremen	21 (0; 3)	11 (3; 5)	96	15	252
Hamburg	15 (4; 5)	5 (4; 4)	157	30	274
Hesse	58 (7; 11)	14 (5; 9)	797	16	289
Mecklenburg-Western Pomerania	22 (0; 0)	13 (3; 6)	259	4	88
Lower Saxony	84 (2; 7)	35 (11; 16)	1436	11	139
North Rhine-Westphalia	188 (23; 29)	95 (60; 74)	2217	21	277
Rhineland-Palatinate	25 (2; 3)	10 (8; 8)	462	17	236
Saarland	7 (0; 1)	4 (2; 2)	158	23	203
Saxony	26 (1; 1)	13 (11; 12)	690	10	333
Saxony-Anhalt	33 (0; 0)	16 (11; 8)	449	6	154
Schleswig-Holstein	28 (1; 0)	14 (4; 6)	565	8	92
Thuringia	18 (4; 1)	6 (2; 5)	352	10	232
total/Germany	824 (91; 132)	385 (206; 274)	11,317	21	258

^a^ [24]; ^b^ Cumulative cases per 100 Residents in NHs according to official statistics—nominator: daily report of the RKI from 5 May 2020 [25] and from 19 January 2021 [26]; Denominator: total number of all NH residents for December 2019 in the federal state [24]).

**Table 2:** Sample Description.

		1st Wave				2nd Wave			
**Outcome**									
		**NHs with Cases**		**Number of Cases**		**NHs with Cases**		**Number of Cases**	
		N	*n* (%)	N	Mean (sd)	N	*n* (%)	N	Mean (sd)
residents		787	91 (11.6)	91	10.0 (12.4)	370	206 (55.7)	206	20.7 (20.5)
staff		759	132 (17.4)	132	5.4 (7.2)	371	272 (73.3)	272	11.0 (12.5)
residents (deaths)		765	49 (6.4)	49	5.3 (5.3)	350	132 (37.7)	132	7.3 (6.7)
**NH Characteristics**									
		N	Mean (sd)			N	Mean (sd)		
Facility size		782	90.6 (42.2)			379	86.8 (40.6)		
CRR		739	0.62 (0.17)			346	0.61 (0.24)		
		N	*n* (%)			N	*n* (%)		
Ownership	non-profit	807	520 (64.4)			368	229 (62.2)		
	for-profit	807	287 (35.6)			368	139 (37.8)		
Measure-ments	IPC training	688	624 (90.7)			n.c. ^a^	-		
	monitoring	691	570 (82.5)			n.c. ^a^	-		
	absenteeism	700	679 (97.0)			n.c. ^a^	-		
	testing staff	n.c. ^a^	-			376	daily	weekly	irregularly
							148 (39.4)	223 (59.3)	5 (1.3)
	testing visitors	n.c. ^a^	-			368	always ^b^		not always
							304 (82.6)		64 (17.4)
	lack of testing	n.c. ^a^	-			377	yes ^c^		no
							116 (30.8)		261 (69.2)
**Type of NH**									
Speciali-sation	none	824	694 (84.2)			373	291 (78.0)		
	dementia	824	86 (10.4)			373	64 (17.2)		
	other	824	44 (5.3)			373	18 (4.8)		
Short-term care provision		681	432 (63.4)			330	226 (68.5)		
**Environment**									
General spread	below average	819	341 (41.6)			381	125 (32.8)		
	above average	819	478 (58.4)			381	256 (67.2)		

^a^ n.c. = not collected; ^b^ always before entering the facility; ^c^ tests could not be carried out as planned.

**Table 3:** Zero models multiple.

Covariate	Cases among Residents (1st Wave N = 709, 2nd Wave N = 322)		Cases among Staff (1st Wave N = 688, 2nd Wave N = 324)		Deaths among Residents (1st Wave N = 694, 2nd Wave N = 304)	
	OR	95% CI	OR	95% CI	OR	95% CI
1st Wave						
Facility size	1.007 **	1.002–1.013	1.005 *	1.001–1.010	1.00	0.99–1.01
CRR	4.12 *	1.01–16.82	4.35 *	1.26–14.97	16.33 **	2.88–92.50
Ownership (for-profit vs. non-profit)	0.60	0.33–1.09	0.69	0.42–1.13	1.09	0.52–2.31
Monitoring vs. no monitoring	8.04 **	1.93–33.60	4.00 **	1.68–9.50	8.98 *	1.21–66.68
Monitoring missing vs. no monitoring	5.17 *	1.02–26.22	3.31 *	1.13–9.73	5.09	0.51–50.95
Short-term care provsion vs. no short-term care provision	1.30	0.72–2.35	1.64	0.98–2.73	-	-
Short-term care missing vs. no short-term care provision	1.52	0.66–3.48	1.68	0.81–3.48	-	-
General spread (above-average vs. below-average)	2.40 **	1.33–4.35	2.76 ***	1.66–4.58	9.42 ***	2.81–31.54
2nd Wave						
Facility size	1.02 ***	1.01–1.03	1.02 ***	1.01–1.03	1.013 ***	1.006–1.021
CRR	2.14	0.50–9.13	5.86 *	1.08–31.91	3.96	0.84–18.75
Ownership (for-profit vs. non-profit)	0.40 ***	0.24–0.67	0.43 **	0.24–0.77	0.60	0.34–1.04
Lack of testing vs. no lack of testing	1.73 *	1.01–2.96	2.08 *	1.06–4.09	1.64	0.97–2.77
General spread (above average vs. below average)	1.23	0.72–2.11	3.48 ***	1.95–6.21	1.82	1.00–3.31

OR odds ratio; CI confidence interval; significance levels: * *p* < 0.05; ** *p* < 0.01; *** *p* < 0.001.

**Table 4:** Count models multiple.

Covariate	Cases among Residents (1st Wave N = 709, 2nd Wave N = 322)		Cases among Staff (1st Wave N = 688, 2nd Wave N = 324)		Deaths among Residents (1st Wave N = 694, 2nd Wave N = 304)	
	est.	95% CI	est.	95% CI	est.	95% CI
1st Wave						
Facility size	−0.002	−0.01–0.01	0.007	−0.003–0.02	0.005	−0.01–0.02
CRR	7.23 ***	3.71–10.74	4.93 ***	2.09–7.76	3.79 *	0.16–7.42
Ownership (for-profit vs. non-profit)	0.79	−0.66–2.25	0.83	−0.57–2.22	0.39	−0.55–1.34
Monitoring vs. no monitoring	2.22	−0.68–5.13	1.95 *	0.21–3.69	0.05	−2.65–2.74
Monitoring-missing vs. no monitoring	2.29	−1.38–5.96	−1.63	−4.05–0.78	−0.02	−2.98–2.95
Short-term care provsion vs. no short-term care provision	−0.51	−1.65–0.63	−0.51	−1.62–0.60	-	-
Short-term care-missing vs. no short-term care provision	0.15	−1.52–1.83	−1.01	−2.36–0.34	-	-
General spread (above-average vs. below-average)	1.67 **	0.49–2.85	1.67 *	0.35–2.98	−0.39	−1.98–1.20
AIC null model	1116		1316		617	
AIC	967		1147		548	
2nd Wave						
Facility size	0.008 **	0.002–0.014	0.011 ***	0.006–0.016	0.007 **	0.002–0.012
CRR	1.35 *	0.04–2.67	1.03	0.00–2.07	1.42 *	0.21–2.63
Ownership (for-profit vs. non-profit)	0.32	−0.16–0.80	−0.08	−0.46–0.31	0.10	−0.36–0.56
Lack of testing vs. no lack of testing	−0.04	−0.44–0.36	0.07	−0.27–0.40	−0.13	−0.52–0.26
General spread (above average vs. below average)	0.22	−0.27–0.70	−0.01	−0.42–0.41	−0.03	−0.55–0.50
AIC null model	2139		2237		1240	
AIC	1837		1916		1043	

est. estimate; CI confidence interval; significance levels: * *p* < 0.05; ** *p* < 0.01; *** *p* < 0.001.

**Figure A1:**
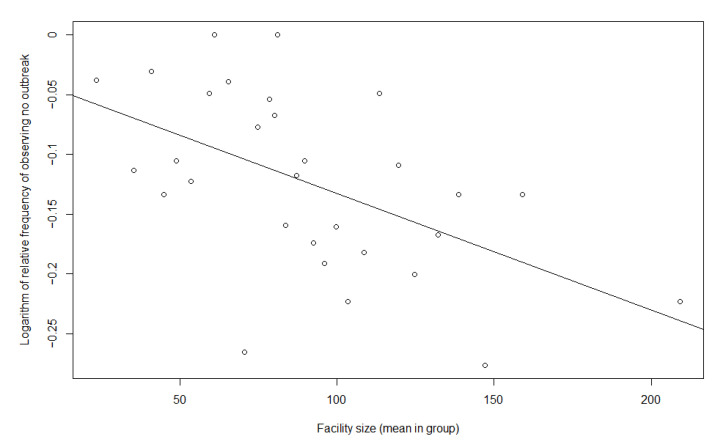
Plotting the logarithmic frequency of observing no outbreak against the facility size in the first wave.

**Figure A2:**
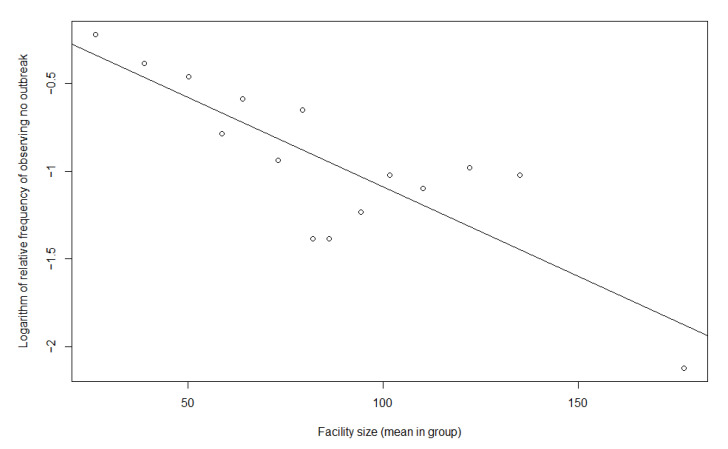
Plotting the logarithmic frequency of observing no outbreak against the facility size in the second wave.

## Data Availability

The data presented in this study are not publicly available due to privacy regulations. For more information about the survey and the data, please contact the corresponding author.

## Appendix A

**Table A1:** Significant variables concerning the count and the zero model.

Covariate (Reference Category)	Cases among Residents				Cases among Staff				Deaths among Residents			
	1st Wave		2nd Wave		1st Wave		2nd Wave		1st Wave		2nd Wave	
	zero	count	zero	count	zero	count	zero	count	zero	count	zero	count
CRR	↗	↗	-	↗	↗	↗	↗	-	↗	↗	-	↗
Ownership (profit vs. non-profit)	↘	-	↘	-	↘	-	↘	-	↘	-	↘	-
Monitoring vs. no monitoring	↗	-	n.c.	n.c.	↗	-	n.c.	n.c.	↗	-	n.c.	n.c.
Lack of testing vs. no lack of testing	n.c.	n.c.	↗	-	n.c.	n.c.	↗	-	n.c.	n.c.	↗	-
Short-term care provsion vs. no short-term care provision	↗	-	-	-	↗	-	-	-	-	-	-	-
General spread (above-average vs. below-average)	↗	-	↗	-	↗	↗	↗	-	↗	-	↗	-

n.c. = not collected; ↗ = significant positive impact; ↘ = significant negative impact; - = no significant impact (significance level of 0.05).
